# Purification and characterization of α-amylase from *Trichoderma pseudokoningii*

**DOI:** 10.1186/s12858-018-0094-8

**Published:** 2018-06-14

**Authors:** Wesam H. Abdulaal

**Affiliations:** Biochemistry Department, Faculty of Science, King Abdulaziz University, Jeddah, Kingdom of Saudi Arabia

**Keywords:** α-Amylase, *Trichoderma pseudokoningii*, Purification, Characterization

## Abstract

**Background:**

Previous studies have demonstrated that members of *Trichoderma* are able to generate appreciable amount of extracellular amylase and glucoamylase on soluble potato starch. In this study the α-amylase was purified and characterized from *Trichoderma pseudokoningii* grown on orange peel under solid state fermentation (SSF).

**Results:**

Five α-amylases A1-A5 from *Trichodrma pseudokoningii* were separated on DEAE-Sepharose column. The homogeneity of α-amylase A4 was detected after chromatography on Sephacryl S-200. α-Amylase A4 had molecular weight of 30 kDa by Sephacryl S-200 and SDS-PAGE. The enzyme had a broad pH optimum ranged from 4.5 to 8.5. The optimum temperature of A4 was 50 °C with high retention of its activity from 30 to 80 °C. The thermal stability of A4 was detected up to 50 °C and the enzyme was highly stable till 80 °C after 1 h incubation. All substrate analogues tested had amylase activity toward A4 ranged from 12 to 100% of its initial activity. The Km and Vmax values of A4 were 4 mg starch/ml and 0.74 μmol reducing sugar, respectively. The most of metals tested caused moderate inhibitory effect, except of Ca^2+^ and Mg^2+^ enhanced the activity. Hg^2+^ and Cd^+ 2^ strongly inhibited the activity of A4. EDTA as metal chelator caused strong inhibitory effect.

**Conclusions:**

The properties of the purified α-amylase A4 from *T. pseudokoningii* meet the prerequisites needed for several applications.

## Background

α-Amylases are produced by plants, animals and a wide variety of bacteria and fungi. Microbial sources of α-amylases are cost effective and appropriate for industrial demands [1–5]. Amylases have the major world market share of enzymes in several applications. Amylases are used in industrial processes like detergents, food, textiles and paper. They can also use for pharmaceutical and fine chemical [6, 7]. Interestingly, it was reported that α-amylase is composed in around 90% of all liquid detergents. Moreover, the usage of α-amylases in detergents used for automatic dishwashing is increased [8].

Several works has been performed to improve the efficient utilization of agro-industrial residues in order to produce enzymes from microorganism which have commercial importance [9, 10]. Previous studies have demonstrated that members of *Trichoderma* are able to generate appreciable amount of extracellular amylase and glucoamylase on soluble potato starch [11, 12]. The susceptibility of starch to amylase attack depends on the properties of the specific starch, such as e.g. degree of gelatinization, and the characteristics of the specific amylase [13]. Therefore, in this study the α-amylase was purified and characterized from *Trichoderma pseudokoningii* grown on orange peel under solid state fermentation.

## Methods

### Trichoderma pseudokoningii

*Trichoderma pseudokoningii* was provided from Plant Pathology Unit, National Research Centre, Cairo, Egypt.

### Solid-state fermentation

Two g dried orange peel was soaked with 2 ml distilled water in 25 ml Erlenmeyer flask and autoclaved. *T. pseudokoningii* was inoculated in the flask and incubated at 28 °C for seven days.

### Purification of α-amylase

*T. pseudokoningii* α-amylase was extracted in distilled water and centrifuged at 12,000 rpm for 10 min. The supernatant (crude extract) was dialyzed against 20 mM Tris-HCl buffer, pH 7.2. The crude extract was loaded on a DEAE- Sepharose column (12 × 1.6 cm i.d.). Five protein peaks were separated with different concentrations of NaCl (0.0–0.3 M). These peaks had α-amylase activity (A1 – A5). A4 with highest activity was loaded on Sephacryl S-200 column (90 × 1.6 cm i.d.).

### α-Amylase assay

α-Amylase activity was measured by determination the liberated reducing sugars as end products according to the method of Nelson [14].

### Protein determination

Bradford [15] method was used to determine the protein.

### M Wt

The M Wt of α-amylase was determined by Sephacryl S-200 and SDS-PAGE [16].

*Characterization of α-amylase.*

### Effect of temperature

At temperature range of 30-70 °C, the activity of α-amylase was investigated.

### pH optimum

Different buffers were used to examine α-amylase activity at several pH’s.

### Substrate specificity

The enzyme was tested to determination a preference for different substrates such as starch, amylopectin, amylose, glycogen, *β*-cyclodextrin and α- cyclodextrine.

### Metal cations and EDTA

Fe^2+^, Co^2+^, Ca^2+^, Cu^2+^, Ni^2+^, Zn^2+^, Hg^2 +^ and EDTA were incubated with enzyme for 15 prior to substrate addition. One hundred percent of activity has been taken as an enzyme activity without metal ions.

## Results and discussion

**Fig. 1:**
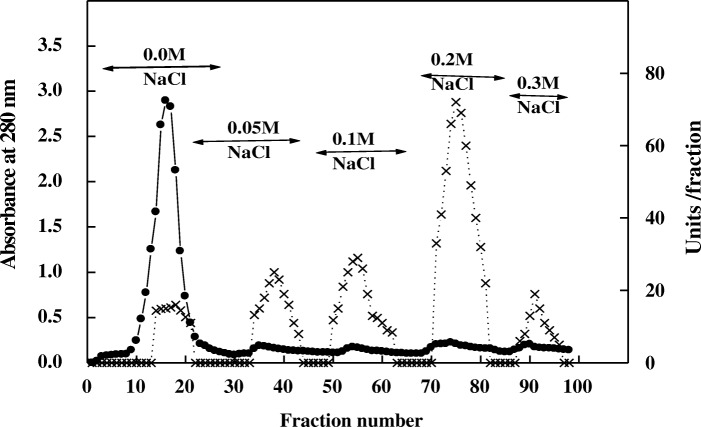
Chromatography of *T. pseudokoningii* α-amylase on DEAE-Sepharose column. (•^___^•) Absorbant at 280 nm, (x ^___^ x) units/fraction

**Fig. 2:**
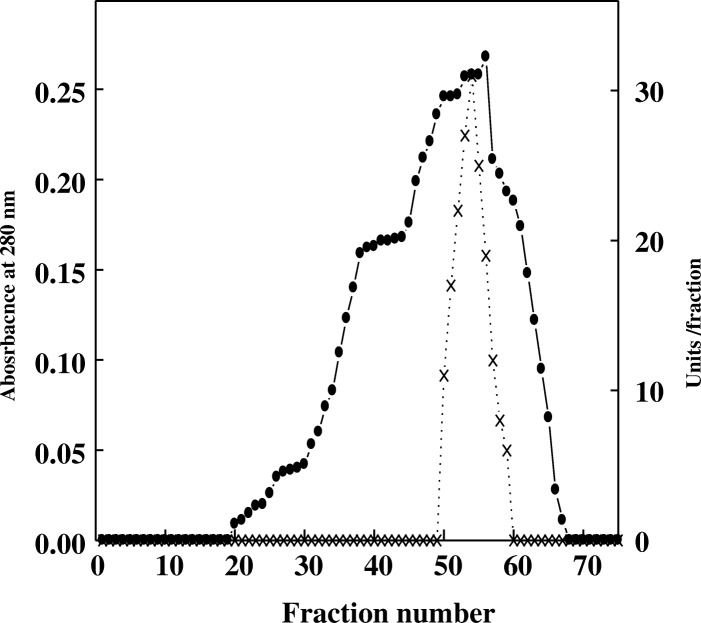
Chromatography of *T. pseudokoningii* α-amylase A4 DEAE-Sepharose fraction on Sephacryl S-200 column. (•^___^•) Absorbant at 280 nm, (x ^___^ x) units/fraction

**Fig. 3:**
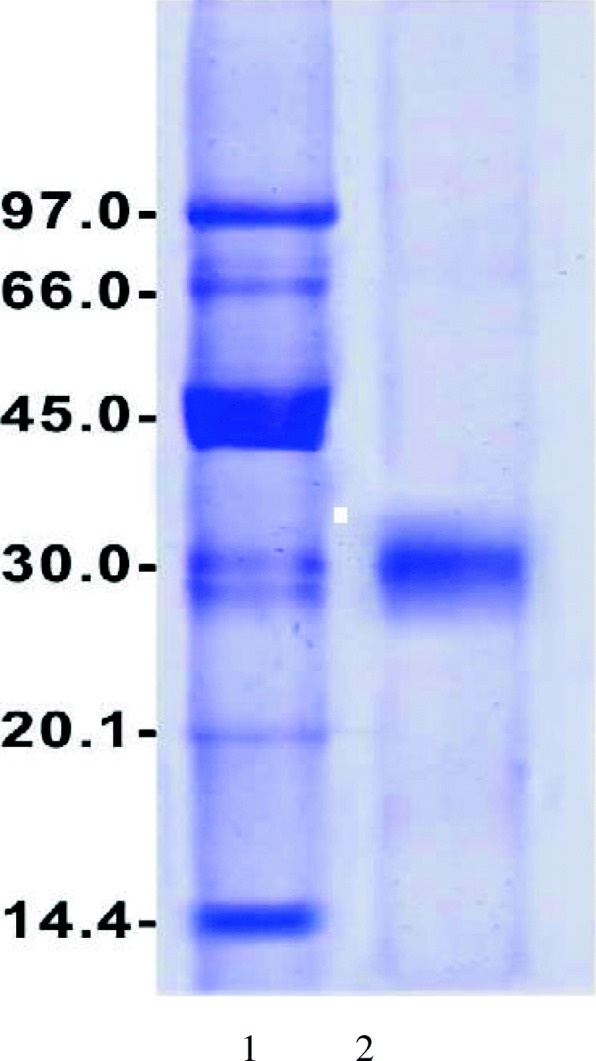
SDS-PAGE for *T. pseudokoningii* α-amylase A4. 1, molecular markers, 2, Sephacryl S-200 A4

Extracellular α-amylase was produced by *T. pseudokoningii* grown on orange peel using solid state fermentation. It was purified by DEAE-Sepharose and Sephacryl S-200 columns. Five α-amylases A1-A5 from *T. pseudokoningii* were separated on DEAE-Sepharose column (Fig. 1). The homogeneity of α-amylase A4 was detected after chromatography on Sephacryl S-200 with specific activity of 550 units/mg protein and fold purification of 15.7 (Figs. 2, 3 and Table 1). α-Amylase A4 had M Wt of 30 kDa by Sephacryl S-200 and SDS-PAGE (Fig. 3). The similar M Wt was detected in *T. matsutake* (34 kDa) [17]. The higher M Wt of α-amylase was detected for *T. harzianum* (70 kDa) [18].

**Table 1:** Purification scheme *for T. pseudokoningii* α-amylase

steps	T. units	T. Protein mg	S.A*	Fold purification	Recovery 100%
Crude extract	605	17.3	34	1	100
Chromatography DEAE-sepharose					
0.0 M NaCl (A1)	17.8	6.8	2.6	0.07	2.9
0.05 M NaCl(A2)	28	0.39	71.8	2.05	4.6
0.1 M NaCl (A3)	31	1	31	0.88	5.1
0.2 M NaCl (A4)	422	1.04	405.7	11.6	69
0.3 M NaCl (A5)	25	1.9	13	0.37	4.1
Sephacryl S-200 A4	110	0.2	550	15.7	18

S.A*: Specific activity (total units/ mg protein)

**Fig. 4:**
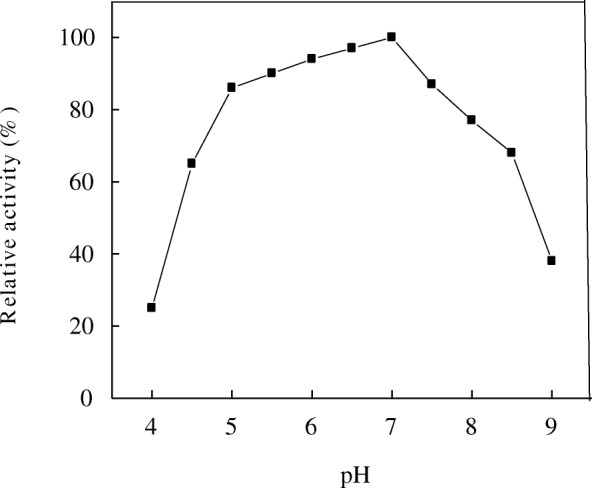
Optimum pH of *T. pseudokoningii* α-amylase A4

The hydrogen ion concentration is one of the most fundamental factors affecting the enzymatic activity. Fig. 4 shows the maximal activity of A4, which had a broad pH optimum ranged from pH 4.5 to 8.5. α-Amylases from different fungi had sharp pH optima ranged from acidity to alkalinity [19–22].

**Fig. 5:**
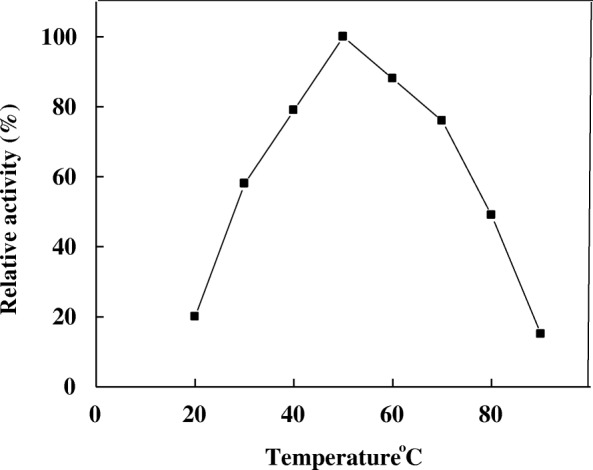
Optimum temperature of *T. pseudokoningii* α-amylase A4

**Fig. 6:**
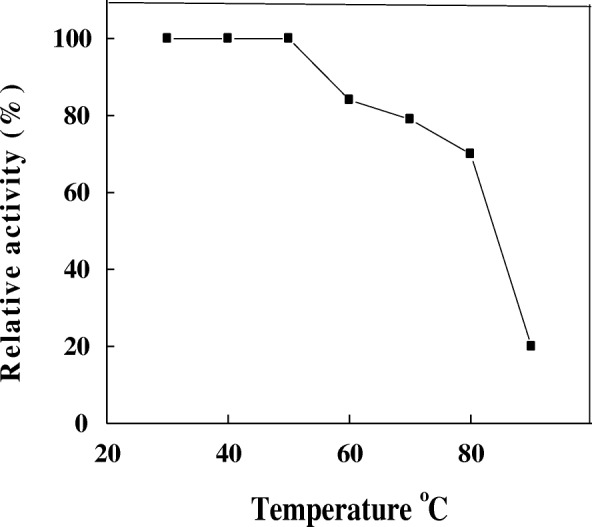
Thermal stability of *T. pseudokoningii* α-amylase A4

Maximal activity of A4 was recorded at 50 °C with high retention of its activity from 30 to 80 °C (Fig. 5). At 20 and 90 °C the enzyme lost the most of its activity. Optimum temperature of α- amylase isolated from several microorganisms was obtained at temperatures ranging from 45 °C to 65 °C [8, 21–23]. The thermal stability of A4 was detected up to 50 °C and the enzyme was highly stable till 80 °C after 1 h incubation (Fig. 6). Similarly, *T. harzianum* α-amylase had thermal stability up to 50 °C [11].

**Fig. 7:**
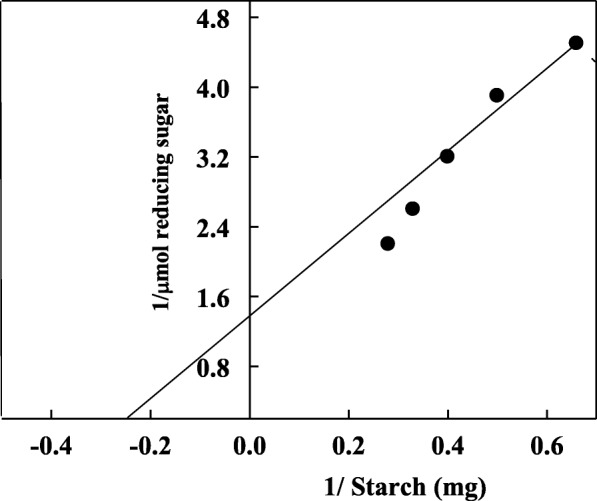
linewever burkplot of *T. pseudokoningii* α-amylase A4 using different concentration of starch

The substrate specificity was studied to evaluate the ability of A4 to catalyze the hydrolysis of some analogues structurally related to starch. Table 2 shows that A4 catalyzed the hydrolysis of substrates in the order of starch < glycogen < amylopectin < amylose < α- cyclodextrin < β-cyclodextrin. These results indicating that the substrates with high molecular weight had high affinity toward enzyme. de Azevedo et al. [11] showed that α-amylase of *T. harzianum* degraded the starch, glycogen and amylopectin but not cellobiose, α-cyclodextrin or β-cyclodextrin. The starch and amylopectin were the substrates preferentially hydrolyzed by *A. tamarii* α-amylase [24]. In a decreasing order, *A. sporosulcatum* α-amylase catalyzed the degradation of starch > glycogen > dextrin [25]. The Km and Vmax values of A4 were 4 mg starch/ml and 0.74 μmol reducing sugar, respectively (Fig. 7). Similarly, the K_m_ values of α-amylases from *T. harzianum* (3.5 mg/ml) [11] and *Geobacillus thermodenitrificans* (3.05 mg /ml) were detected [19].

**Table 2:** Substrate specificity of *T. pseudokoningii* α-amylase A4

Substrate	Relative activity %
Potato starch	100
Glycogen	85
Amylopectin	73
Amylose	55
α-Cyclodextrin	13
β-Cyclodextrin	12

Many of amylases had Ca^2+^ cation in active site, therefore they are metal ion-dependent [26, 27]. The most of metals tested caused moderate inhibitory effect, except of Ca^2+^ and Mg^2+^ enhanced the activity of A4. Hg^2+^ and Cd^+ 2^ strongly inhibited the activity of A4 (Table 3). The similar results were detected in α-amylase *T. harzianum* concerning the effect of metal ions [11]. The A4 can be used as a biosensor for heavy metals Hg^2+^ and Cd^2+^ as its activity is greatly affected by the two and an enzymatic biosensor can be most sensitive and specific. EDTA as metal chelator caused strong inhibitory effect on activity of A4 suggesting that the active site of the enzyme contained metal ions.

**Table 3:** Influence of metal and EDTA at 5 mM on *T. pseudokoningii* α-amylase A4

Metal cations	Relative activity %
Control	100
Ca^2+^	152
Mg^2+^	134
Ni^2+^	100
Pb^2+^	80
Co^2+^	72
Hg^2+^	38
Cu^2+^	75
Zn^2+^	65
Cd^2+^	37
EDTA	28

## Conclusions

In the present study, the purified α-amylase A4 from *T. pseudokoningii*, grown on agro-industrial residues under SSF, characterized by broad pH optima, thermal stability, all substrate analogues tested had amylolytic activity and moderate tolerance towards some metal ions. Similarly, α-amylases produced from *Bacillus licheniformis, Bacillus stearothermophilus,* and *Bacillus amyloliquefaciens* show broad pH optimum and thermal stability and promising potential in a number of industrial applications in processes such as food, fermentation, textiles and paper industries [28, 29].These findings make α-amylase A4 useful in the several applications.

## Abbreviations

A: α-Amylase
DEAE: Diethylaminoethyl
EDTA: Ethylenediaminetetraacetic acid
SDS-PAGE: Sodium dodicylsulphate-poly acrylamide gel electrophoresis
